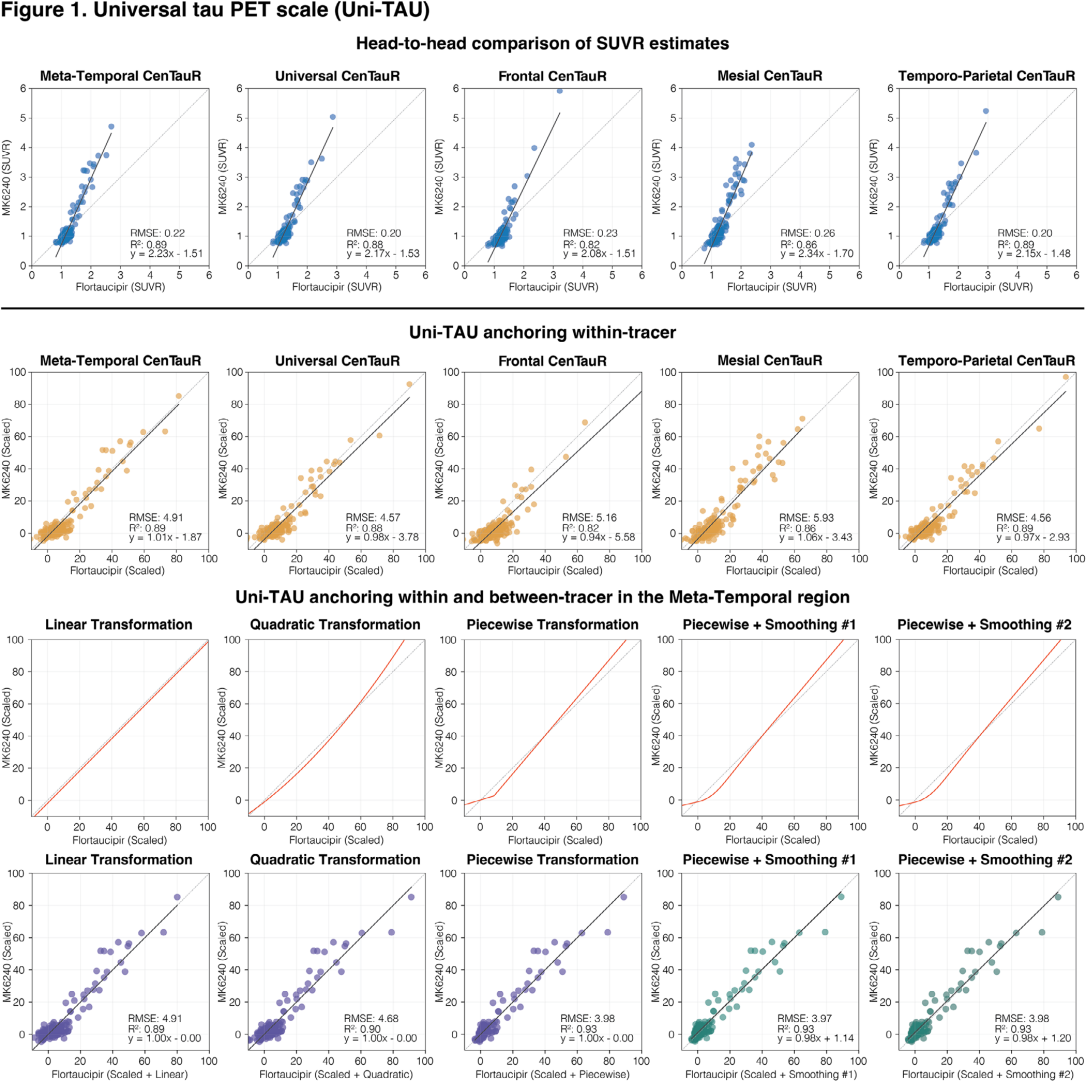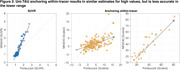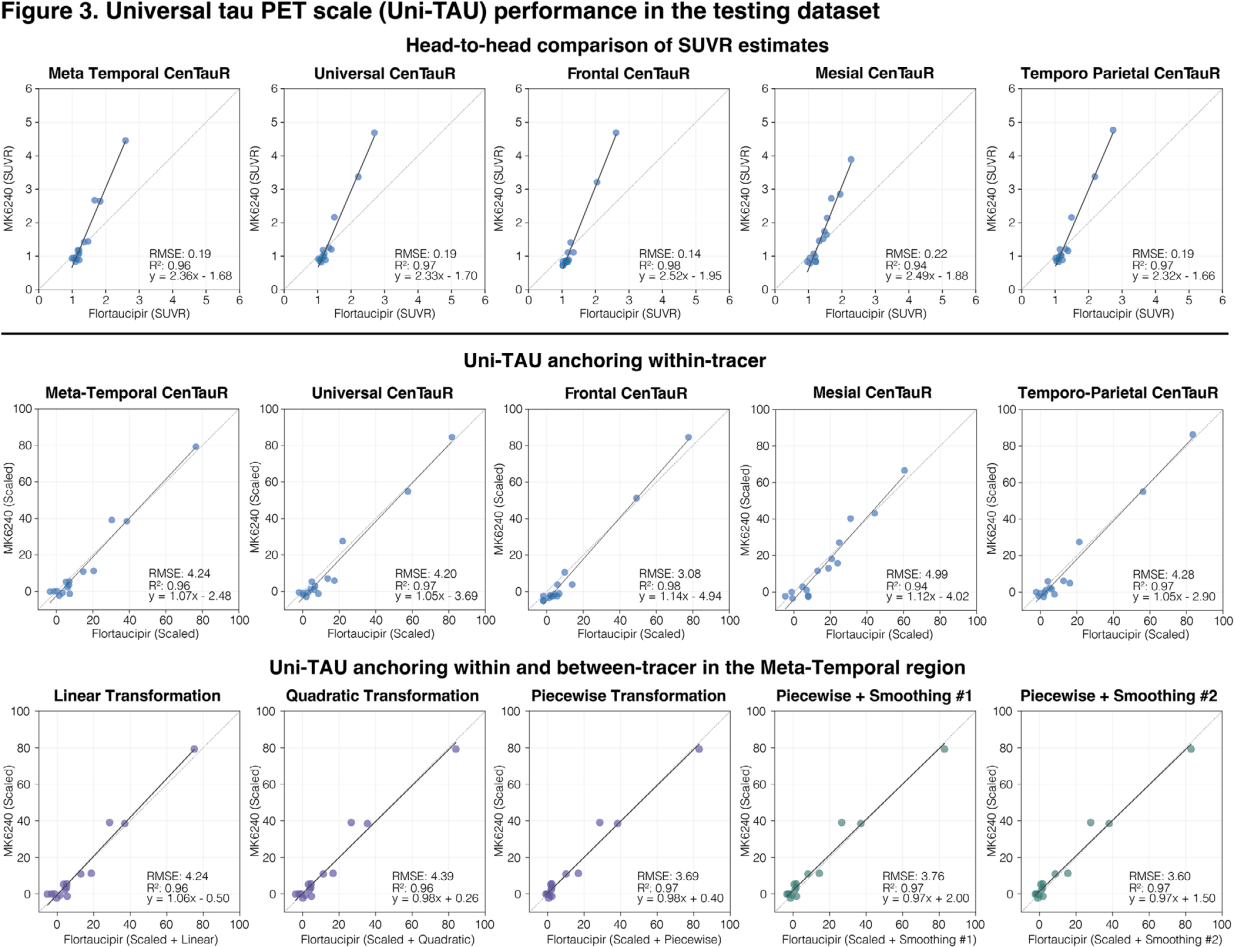# Universal tau PET scale (Uniτ) – The HEAD Study

**DOI:** 10.1002/alz.089555

**Published:** 2025-01-09

**Authors:** Guilherme Povala, Guilherme Bauer‐Negrini, Bruna Bellaver, Firoza Z Lussier, Livia Amaral, Pamela C.L. Ferreira, Bruno Zatt, Dana Tudorascu, Brian J Lopresti, William J. Jagust, William E Klunk, Val J. Lowe, David N. soleimani‐meigooni, Hwamee Oh, Belen Pascual, Brian A. Gordon, Pedro Rosa‐Neto, Tharick A. Pascoal

**Affiliations:** ^1^ University of Pittsburgh, Pittsburgh, PA USA; ^2^ Universidade Federal de Pelotas, Pelotas Brazil; ^3^ Lawrence Berkeley National Laboratory, Berkeley, CA USA; ^4^ Department of Radiology, Mayo Clinic, Rochester, MN USA; ^5^ Memory and Aging Center, Weill Institute for Neurosciences, University of California, San Francisco, San Francisco, CA USA; ^6^ Brown University, Providence, RI USA; ^7^ Houston Methodist Research Institute, Houston, TX USA; ^8^ Washington University in St. Louis School of Medicine, St. Louis, MO USA; ^9^ Translational Neuroimaging Laboratory, The McGill University Research Centre for Studies in Aging, Montréal, QC Canada

## Abstract

**Background:**

The HEAD study aims to collect a large dataset of multiple tau‐PET tracers to provide robust anchor values for tau‐PET harmonization. Here, we tested the hypothesis that anchoring two tau tracer uptake values using head‐to‐head measurements has the potential to generate an accurate universal tau‐PET scale, named Uniτ(tau).

**Methods:**

We assessed 200 individuals across the aging and AD spectrum (Training: HEAD data freeze 2.0, n=185; Testing: UPitt dataset (Gogola et al.), n=15) with [^18^F]Flortaucipir and [^18^F]MK‐6240 tau‐PET. SUVRs were processed to a common 8mm FWHM, with inferior cerebellar gray matter as the reference region (Pascoal et al.). Uniτ explored two anchoring/harmonization methods. First, we examined within‐tracer anchoring by creating anchor values based on the mean SUVR of Youngs (<25 years) and 95th percentile voxels from cognitively impaired individuals. Second, we explored within‐ plus between‐tracer anchoring, employing linear (e.g., piecewise) and non‐linear regressions. To address the inherent problem of discontinuity of piecewise transformations, we implemented two smoothing methods at the inflection point between equations, transforming them into a continuous function.

**Results:**

Uniτ scale anchoring within‐tracer resulted in similar estimates for high values, but less accurate in the lower range (Figure 1,2). Anchoring within‐ plus between‐tracer improved estimate consistency, with the piecewise transformation generating the best results. The piecewise smoothing equation yielded estimates comparable to those obtained from the piecewise method without smoothing. This allowed for the use of a single formula. In addition, this leads to more robust results when the goal is to study longitudinal changes in the scale (data not shown). UPitt testing dataset showed similar results to the training set (Figure 3).

**Conclusion:**

Our preliminary findings suggest that anchoring tau‐PET values both within and between tracers has the potential to harmonize tau‐PET tracers, while preserving their characteristics. Currently, piecewise smoothing is the preferred method for Uniτ, but we are continuously fine‐tuning scale parameters as we acquire more data. The final scale parameters will be determined based on extensive training and testing data from multiple tracers. This cautious methodology holds the promise of delivering reliable, robust, and reproducible results, ensuring safe usage of the scale in clinical trials, and potentially paving the way for future use in clinical practice.